# Tonsilitis

**Published:** 1903-11

**Authors:** 


### TONSILITIS.

Sod. benzoate .................................. 1 oz.
Ex. cascara sagrad., fl. ........................ 1 oz.
Tongaline ................ q. s. ad. ............... 6 ozs.

M. Sig.—A teaspoonful every two to four hours.

